# The burden and scope of childhood cancer in displaced patients in Jordan: The King Hussein Cancer Center and Foundation Experience

**DOI:** 10.3389/fonc.2023.1112788

**Published:** 2023-03-24

**Authors:** Rawad Rihani, Sima Jeha, Mayse Nababteh, Carlos Rodriguez-Galindo, Asem Mansour, Iyad Sultan

**Affiliations:** ^1^ Department of Pediatrics, King Hussein Cancer Center, Amman, Jordan; ^2^ Department of Global Pediatric Medicine, St. Jude Children’s Research Hospital, Memphis, TN, United States; ^3^ International Fundraising and Development Department, King Hussein Cancer Foundation, Amman, Jordan

**Keywords:** Syria, Jordan, refugees, humanitarian response, childhood cancer, displaced children, access to health care, public health

## Abstract

**Introduction:**

Jordan hosts one of the highest numbers of refugees per capita in the world, with the Syrian crisis leading to an influx of displaced persons to the already vulnerable population. However, limited resources and a lack of cancer-care strategies have made it difficult for refugees in Jordan to access quality cancer care. The King Hussein Cancer Center (KHCC) and Foundation (KHCF) have played a pivotal role in providing financial and medical support for displaced children with cancer, treating 968 non-Jordanian children with cancer between 2011-2022, with a median age of 6 years. Of these, 84% were fully funded by KHCF, and nationalities included Syrians (29%), Palestinians (26%), Iraqis (23%), and Yemenis (17%). Cancer diagnoses included solid tumors (44%), leukemia (23%), lymphoma (13%), bone sarcomas (9.5%), and retinoblastoma (9.1%). The median cost of treatment was JOD 18,000 (USD 25,352), with a total estimated cost of JOD 23.8 million (USD 33.5 million). More recently, in partnership with St. Jude Children’s Research Hospital (SJCRH), two successive humanitarian funds (HF) were established to optimize cancer care for displaced children in Jordan.

**Results:**

Between February 2018 and September 2022, 51 children were fully treated on KHCC-SJCRH-HF, with a median age of 6 years and nationalities including Syrians (80%), Iraqis (6%), and Yemenis (8%). The most common cancer diagnoses were leukemia (41%), lymphoma (25%), solid tumors (24%), retinoblastoma (6%), and brain tumors (4%). Of these, 94% are alive and 51% are still receiving coverage. The median coverage for patients was JOD 21,808 (USD 30,715), and the total cost of treatment on KHCC/KHCF-SJCRH/American Lebanese Syrian-Associated Charities HF1 and HF2 was JOD 1.44 million (USD 1.97 million) and JOD 1.18 million (USD 1.67 million), respectively.

**Conclusion:**

This experience highlights the high burden of displaced children with cancer in Jordan, and the importance of local foundations like KHCC/KHCF and partnerships with international partners like SJCRH in providing lifesaving humanitarian initiatives and quality cancer care. Innovative cancer-care delivery models and sustainable financing are essential to ensure continuous coverage and access to cancer care for displaced persons in Jordan.

## Introduction

Refugees constitute a population with unique vulnerabilities, and addressing their needs requires extensive support and places substantial burdens on hosting countries and the global community. Over the past decades, the number of refugees seeking refuge has been on a steady increase (1). Conflicts in countries such as Iraq, Palestine, and Yemen have led to increased displacement in urban areas. In 2011, the Syrian crisis had a profound impact on neighboring countries, many of which were unprepared for the scale of the situation (2).

Jordan, a country with a population of 11 million located in the Middle East and bordered by Syria to the north, has extensive experience in handling refugee crises and has well-established mechanisms in place to maintain the wellbeing of refugees within the country. Although considered by the World Bank as an upper-middle-income country, 13% of the population lives below the poverty threshold (3). Jordan boasts a modernized healthcare system, with a total expenditure per capita for healthcare of 334 US$ (2019), constituting approximately 7.58% of the gross domestic product (1, 3).

Despite its struggling economy, depleted resources, security threats, and enormous sociopolitical burdens, Jordan hosts the second-highest refugee population worldwide, with 89 refugees per 1,000 inhabitants. According to the United Nations High Commissioner for Refugees (UNHCR), 1 in 14 people in Jordan is a refugee (4). Jordan hosts approximately 1.4 million refugees who have escaped conflicts in Syria since 2011. The most recent UNHCR data from 2022 show that Jordan has 762,877 refugees, with 88.7% being Syrians, 8.6% Iraqis, 1.7% Yemenis, and 0.7% Sudanis (5). The actual number of Syrians in Jordan is still debated as there is a persistent gap between the registered number by the UNHCR and the number reported by National Census reports (6). Additionally, many Syrian refugees remain unregistered through the UNHCR. Currently, 82.4% of displaced Syrians reside in the municipal areas of Jordan (5). Additionally, according to the UNHCR, there are approximately 2,242,579 registered Palestinian refugees, in addition to 750,000 to 1 million registered Iraqis who have fled to Jordan before the arrival of Syrian refugees (6), many of whom continue to have restricted access to vital needs, primarily healthcare (1).

Humanitarian assistance focuses on traditional priorities such as the provision of healthcare services to address communicable diseases (7). However, the burden of non-communicable diseases (NCDs) among refugee populations is growing (8), representing substantial emerging challenges in humanitarian responses in both refugee and host communities. The increasing incidence of NCDs among refugee and host communities presents challenges to the provision of quality healthcare (8) as they require more sophisticated diagnostic and management capacities than many communicable diseases. Furthermore, delays in diagnosis and therapy and avoidable complications can result from a lack of access to diagnostic and therapeutic modalities for NCDs (9). The global health and humanitarian community’s response to the increasing incidence of NCDs, including cancer, among millions of refugees fleeing to host countries, has been relatively slow (7). Cancer represents a significant health challenge in refugees and host communities (10, 11) as it is a main cause of mortality, and cancer care delivery requires vigorous and wide-ranging health systems delivering multidisciplinary diagnostic and therapeutic interventions (7, 12). Additionally, for refugees, cancer care necessitates a long-term commitment to the medical system (13).

Jordan’s healthcare system is a mix of public and private healthcare providers, with the Ministry of Health responsible for regulating the public healthcare sector (14). Public healthcare is available to all Jordanian citizens and residents, while private healthcare providers offer high-quality medical care for a fee. The government provides free cancer treatment to all Jordanian citizens, regardless of their socioeconomic status or insurance coverage. The King Hussein Cancer Center (KHCC) in Amman is a world-renowned cancer treatment center that provides state-of-the-art cancer care to patients from Jordan and around the world.

We describe the impact and feasibility of collaborative efforts, the prioritization approach, the strategies of sustenance, and the results of quality cancer care among displaced children treated at the KHCC. Our report reviews our experience in Jordan and gives a deeper understanding into the extent of the humanitarian refugee crisis, pertaining to childhood cancer. It also underscores the need for accurate cancer registration, which will assist in producing the estimates of fiscal resources required to provide quality cancer care and informing humanitarian funding planning and resource allocation. It also highlights the need for upscaling the local and humanitarian efforts to integrate cancer care in their planning.

## Methodology

The KHCC has a robust department of pediatrics that provides fully funded cancer care for Jordanian children. However, since the start of the Syrian crisis in 2011, there has been an influx of displaced cancer patients, particularly Syrian children, seeking treatment at the KHCC. To address the financial needs of these patients, the KHCC and King Hussein Cancer Foundation (KHCF) have established several funds to provide financial assistance for cancer care for displaced children in Jordan. These funds can be applied for by families and by referring physicians.

### King Hussein Cancer Center/King Hussein Cancer Foundation goodwill funds

Goodwill funds (GWFs) were established to provide financial assistance to a considerable number of displaced children with cancer annually. These funds cover the costs of treatment, transportation, accommodations, and food coupons. However, the needs of these patients are ongoing including financial constraints, the disruptions of therapy, and the lack of social support, leaving many displaced patients left behind. The patients eligible for enrollment in these funds are all nationalities of children under 18 years old who are newly diagnosed with cancer and have not received any prior treatment. These funds, however, provide treatment coverage for relapsed/refractory cases ranging from advanced therapy to palliative care. Medical care provided for displaced children at the KHCC is comparable to those applied to Jordanian children. Since the start of the Syrian crisis, the GWFs have been used to cover the costs of cancer care for displaced Syrian children treated at the KHCC through strategic partnerships and focused fundraising efforts.

### King Hussein Cancer Center/King Hussein Cancer Foundation–St. Jude Children’s Research Hospital/American, Lebanese Syrian associated charities humanitarian funds

In response to the growing numbers of displaced children with cancer referrals, the Humanitarian Fund (HF) was established in 2017 as a collaboration between KHCC/KHCF, St. Jude Children’s Research Hospital (SJCRH), and American, Lebanese Syrian Associated Charities (ALSAC). The HF provides funding for a specified number of patients over a period of 2 years, including full treatment for some patients and partial support for others such as diagnostic work-up and surgery. To ensure the sustainability of the program and optimal utilization of funding, eligibility criteria were established, including a documented refugee status, a newly diagnosed curable cancer, commitment to treatment adherence, therapy per standard-of-care, and the provision of follow-up information. The management of this fund is overseen by a joint KHCC/KHCF-SJCRH team, and regular administrative follow-up meetings are held to track the program’s development and adjust as necessary.

Overall, the KHCC and KHCF have established several funds to provide financial assistance for cancer care for displaced children in Jordan, with a focus on, but not limited to, highly curable cancer patients. These funds have been successful in providing treatment for a significant number of patients, and the collaboration with SJCRH/ALSAC has expanded the resources available for this population. However, the needs of these patients are ongoing, and many displaced patients are left behind. Further support is needed to ensure that all displaced children with cancer have access to the care they need.

## Results

### Patient nationalities and enrollment

**Figure 1:**
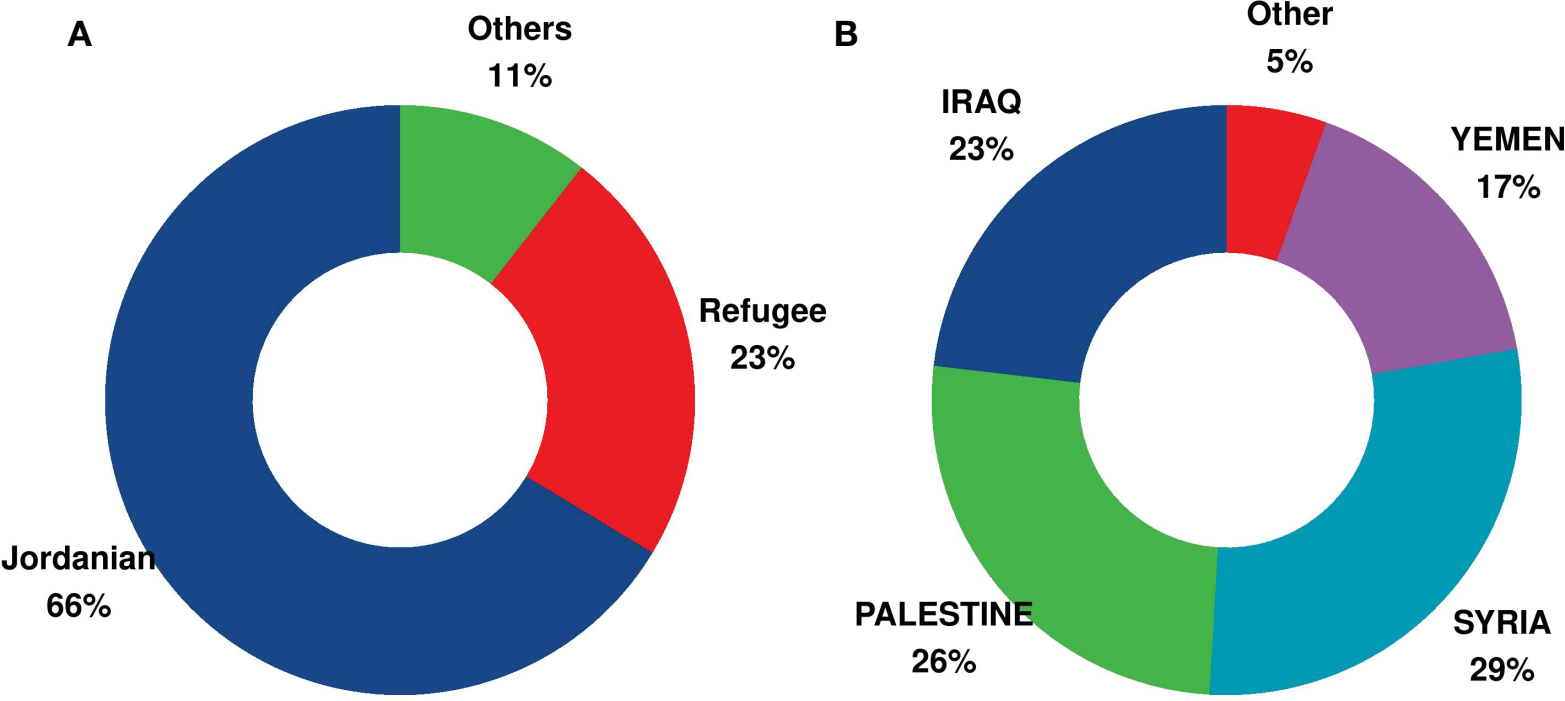
Percentage of Jordanian and non-Jordanian displaced children with cancer treated at the King Hussein Cancer Center (KHCC). **(A)** Percentage of Jordanian and non-Jordanian displaced children with cancer treated at the KHCC and fully registered on the KHCC Cancer Registry for the period 2011–2022. **(B)** Nationalities of the displaced children with cancer treated at the KHCC and fully registered on the KHCC Cancer Registry for the period 2011–2022.

**Figure 2:**
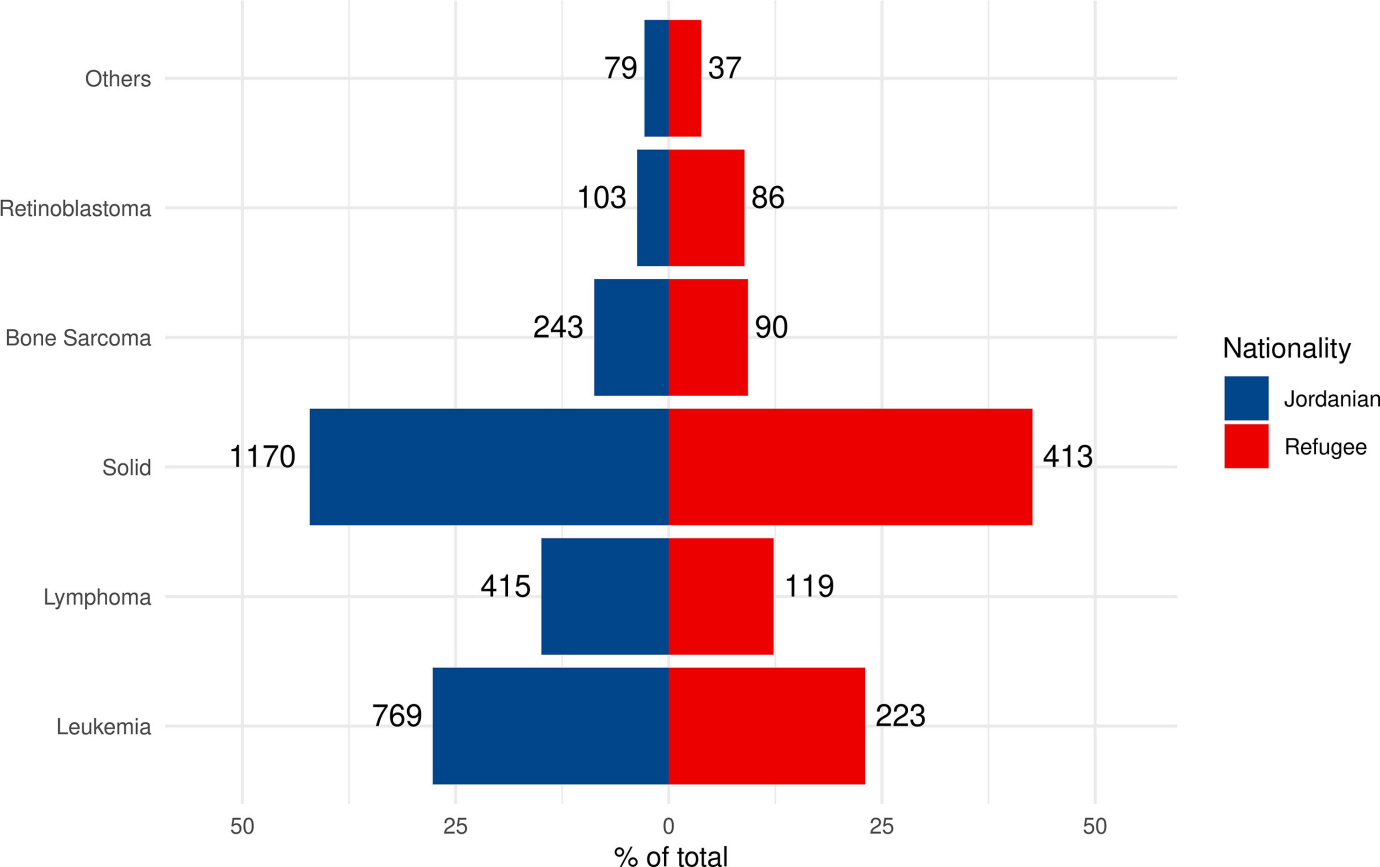
Comparison of the distribution and percentages of cancer diagnosis categories among Jordanian *vs*. non-Jordanian children with cancer treated at the KHCC and fully registered on the KHCC Cancer Registry for the period 2011–2022. Column **(A)** Distribution, frequency, and percentages of childhood cancer categories among Jordanian *vs*. non-Jordanian children with cancer treated at the KHCC and fully registered on the KHCC Cancer Registry for the period 2011–2022. Column **(B)** Distribution, frequency, and percentages of childhood cancer categories among Jordanian *vs*. non-Jordanian children with cancer treated at the KHCC and fully registered on the KHCC Cancer Registry for the period 2011–2022.

**Figure 3:**
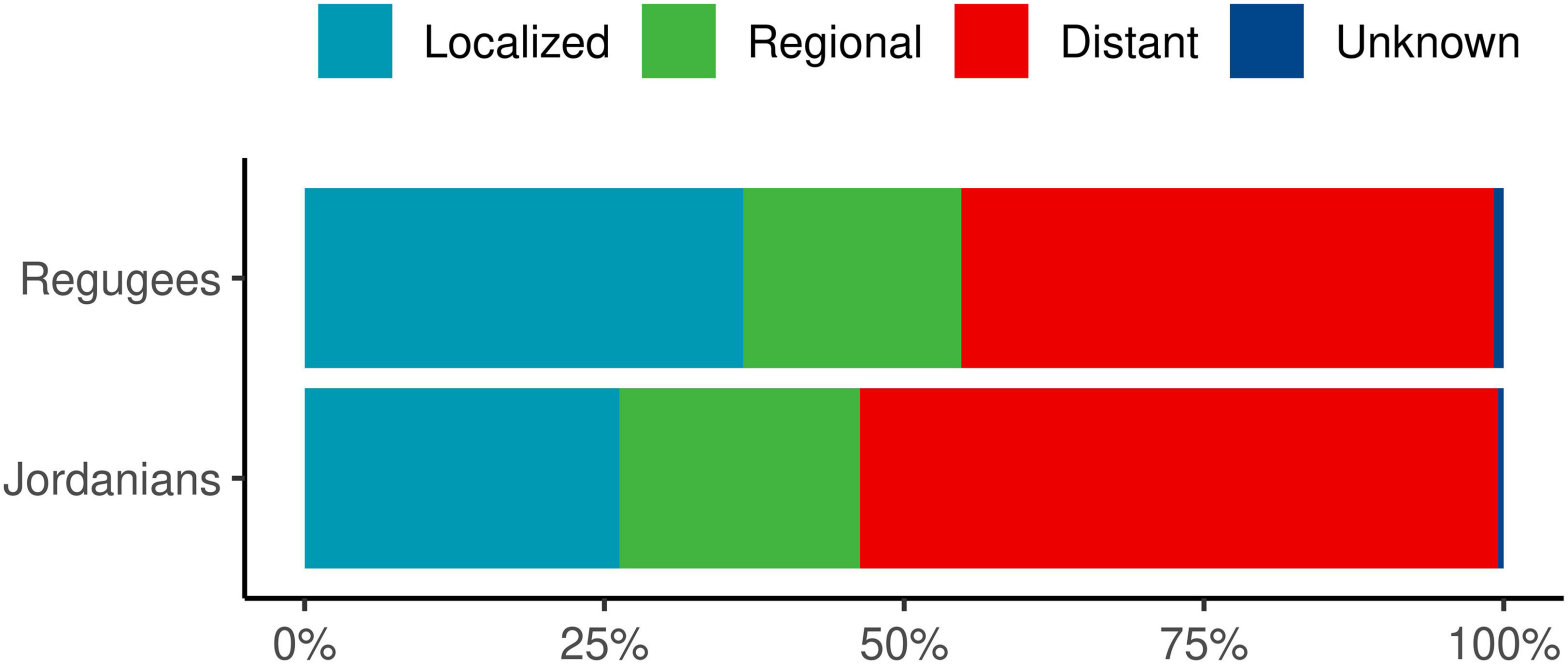
Comparison of the Surveillance, Epidemiology, and End Results (SEER) Histologic Staging of Cancer among Jordanian *vs*. non-Jordanian children with cancer treated at the KHCC and fully registered on the KHCC Cancer Registry for the period 2011–2022. Cancer categories include leukemia*, solid tumors, sarcomas, lymphomas, CNS tumors, and retinoblastomas. *Leukemias are included in distant staging. KHCC, King Hussein Cancer Center; SEER, Surveillance, Epidemiology, and End Results.

Data analysis for the period from 2011 to June 2022 at a median follow-up of 77 months (0.9–146) from the KHCC hospital–based cancer registry (**Supplementary Figure 1**) revealed 2,779 fully registered Jordanian children with cancer (**Figure 1A**), with a median age of 7 years [3, 13 interquartile range (IQR)], and 968 displaced non-Jordanian children (**Figure 1A**) with a median age of 6 years (2,12 IQR). The date of the last follow-up was June 2022. **Figure 1B** describes the displaced patients’ nationalities. Cancer diagnosis and staging for displaced children were similar to Jordanians and include solid tumors (n = 413; 44%), leukemia (n = 223; 23%), lymphoma (n = 119; 13%), bone sarcoma (n = 90; 9.5%), and retinoblastoma (n = 86; 9.1%) (**Figure 2**). The patients’ underlying cancer characteristics are described in **Table 1**. Cancer staging for Jordanian *vs*. displaced children is described in **Figure 3**.

**Table 1:** Patients’ and cancer characteristics among Jordanian and non-Jordanian displaced children with cancer treated at the King Hussein Cancer Center (KHCC).

Characteristic	Jordanian, N (%) N = 2,779*^1^*	Non-Jordanian Displaced Children N (%) N = 968*^1^*
**Age (years)**	7.0 (3.0, 13.0)	6.0 (2.0,12.0)
Gender		
Male	1,532 (55)	555 (57)
Female	1,247 (45)	413 (43)
Displaced Nationalities		
Syrians	———	278 (29)
Palestinians		251 (26)
Iraqi		224 (23)
Yemeni		162 (17)
Others		53 (5)
Cancer Classification		
Bone sarcomas	243 (8.9%)	90 (9.5%)
CNS	9 (0.3%)	2 (0.2%)
Leukemia	769 (28%)	223 (23%)
Lymphoma	415 (15%)	119 (13%)
Retinoblastoma	1,170 (43%)	413 (44%)
Solid tumors	103 (3.8%)	86 (9.1%)
Others	28 (1.0%)	16 (1.7%)
SEER Stage*		
In-situ	0 (0%)	0 (0%)
Localized	653 (26%)	354 (36.6%)
Regional	500 (20%)	176 (18.1%)
Distant	1,324 (53%)	430 (44.4%)
Unknown	12 (0.5%)	8 (0.8%)
Status		
Alive	2,156 (78%)	948 (88%)
Dead	623 (22%)	20 (12%)

* Adolescent and young adults 2020 classification (https://seer.cancer.gov/ayarecode/aya-2020.html).

Data source from the KHCC Cancer Registry for the period 2011–2022.

KHCC, King Hussein Cancer Center; SEER, Surveillance, Epidemiology, and End Results; OS, overall survival; IQR, interquartile range.

The data from KHCF records between 2011 and June 2022 indicate that 809 displaced children were fully covered for cancer treatment. These represented 84% of displaced children registered in our KHCC cancer registry, leaving 159 children who were funded through other sources. Of those covered by the KHCF, 38% (n = 307) still have active coverage, 52% (n = 423) have completed therapy, and 9.4% (n = 76) are deceased. Still, no accurate survival data, however, could be established on non-Jordanian displaced patients as a significant number could not be traced after completing therapy.

Among the non-Jordanian displaced children fully recorded on the KHCC cancer registry, 278 Syrian children were registered (**Table 1**). The patients’ characteristics were compared to 2,779 Jordanian children who were fully treated during the same period (**Table 1**).

### Humanitarian Funds (HF1) and (HF2)

Per the KHCC-SJCRH/ALSAC partnership, two consecutive funding programs were established to provide cancer care for displaced children including a prioritization scheme along with a continuous assessment of resource utilization. The first program (HF1) covered the costs of full cancer treatment for 27 displaced children with cancer (diagnostic evaluations and specific interventions: surgery, radiation, or chemotherapy) over a 2-year period (2018–2020). Once the HF1 funds were consumed, the second program (HF2) was applied to cover another 2-year period (2021–2023). The HF2 program has the same eligibility and is planned steady accrual over a 2-year period; it has enrolled 24 patients to date and is still ongoing.

### King Hussein Cancer Center–St. Jude Children’s Research Hospital HF1 and HF2 patient characteristics

Between February 2018 and September 2022, 51 children with cancer were eligible for KHCC/KHCF-SJCH/ALSAC HFs and were diagnosed and fully treated at the KHCC. Median age, 6 years; IQR,5–9; 65% (n = 33) were males. Nationalities included Syrians (n = 41; 80%), Iraqis (n = 3; 6%), and Yemenis (n = 3; 8%). The most common cancer was leukemia (n = 21; 41%), lymphoma (n = 13; 25%), solid tumors (n = 12; 24%), retinoblastoma (n = 3; 6%) and brain tumors (n = 2; 4%). Of those 94% (n = 45) patients are alive and 51% are still on treatment and receiving coverage.

Because of funding restrictions, off-therapy screening was not sustained beyond the completion of treatment on HF1 and HF2. Further follow-up was covered by the KHCF GWFs. Treatment planning, however, was not impacted by patients’ nationality, and all accrued patients received curative treatment regimens, surgical procedures, and radiation therapy. Follow-up data were collected for all patients who completed therapy or were still receiving first-line treatment on HF1 and 2.

### Cost of treatment of displaced children with cancer

The median coverage (IQR) in JOD and US$ for displaced patients who were fully treated for cancer (n = 809) from KHCF records (between 2011 and June 2022) was 18,000 (5,000, 37,000) and 25,352 (7,042, 52,112), respectively. On the other hand, the median coverage (IQR) in JOD and US$ for displaced patients treated on KHCC/KHCF-SJCRH/ALSAC HF1 and HF2 was 21,808 (10,496–46,522) and 30,715 (14,783,65,523), respectively. With a total cost of treatment on KHCC/KHCF-SJCRH/ALSAC HF1 and HF2 was 1.44 million JOD, US$ 1.97 million, and 1.18 million JOD, 1.67 US$ million—to date), respectively. **Table 2** describes the median cost of treating displaced children with cancer, per diagnosis, at the KHCC (in JOD and US$) between 2011 and 2022.

**Table 2:** Cost of treating displaced children with cancer at the KHCC (in Jordanian dinars and US dollars) between 2011 and 2022.

Diagnosis	Median (IQR) in JOD	Median (IQR) in US$
Leukemia	35,212 (16,345, 69,083)	49,947 (23,184, 97,990)
Lymphoma	25,000 (9,088, 44,562)	35,461 (12,891, 63,208)
CNS	17,000 (7,000, 31,785)	24,113 (9,929, 45,086)
Solids	13,800 (5,000, 28,075)	19,574 (7,092, 39,823)
Retinoblastoma	9,550 (1,457, 17,700)	13,546 (2,067, 25,106)
Bone sarcomas	20,306 (7,500, 51,000)	28,803 (10,638, 72,340)

IQR, interquartile range; JOD, Jordanian dinars; CNS, central nervous system.

### Patient outcomes

**Figure 4:**
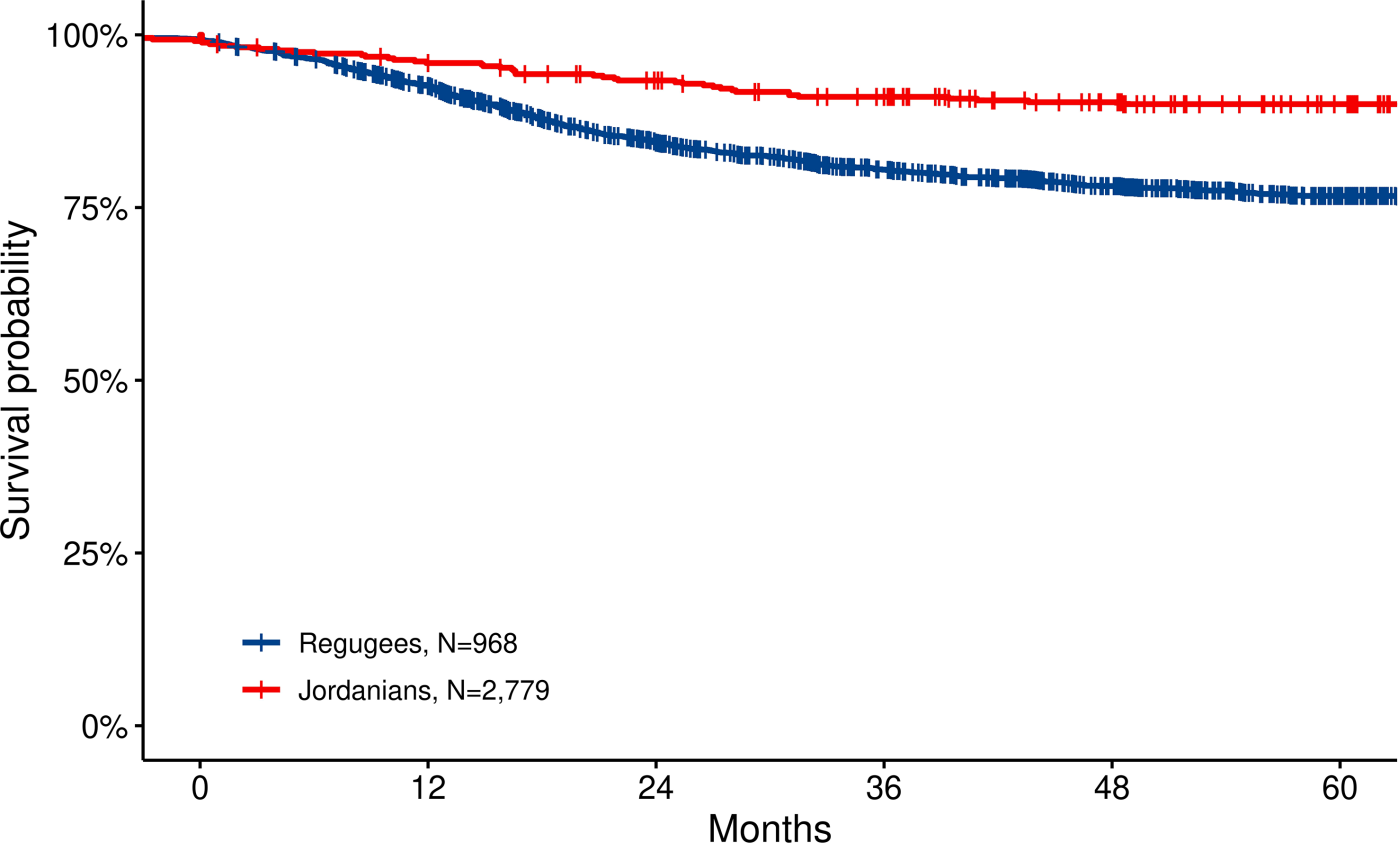
Overall survival for Jordanian and displaced non-Jordanian children with cancer treated at the KHCC and fully registered on the KHCC Cancer Registry for the period 2011–2022. Survival in displaced children with cancer is favorable and comparable to Jordanian children. This can be due to a younger age group, less metastatic patients—or reflecting referral bias, and the possibility that mortality registration is missed for some patients as they do not have a national identification. KHCC, King Hussein Cancer Center.

Kaplan–Meier survival estimates were generated by defining the date of diagnosis as the starting point and death from any cause as the endpoint. Mortality data were sourced from the medical chart or cancer registry records that are linked to national mortality records by social security numbers. While this approach is considered reliable for Jordanians, it may be less accurate for refugees who may have departed from the country or lacked a valid social security number, potentially leading to inflated survival estimates. The estimated 1- and 5-year overall survival of displaced non-Jordanian children are 90.1% +/− 0.6% and 78.9% +/− 0.9%, respectively (**Figure 4**). This outcome was slightly but significantly better (p = 0.037) than Jordanian patients.

## Discussion

Our report highlights the impact and effectiveness of advocacy efforts, partnerships, and financial coverage provided for the treatment of displaced children with cancer in Jordan. The data, collected from the records of the KHCC and KHCF between 2011 and June 2022, highlight the increasing burden of childhood cancer among displaced children in Jordan. The data indicate that comprehensive cancer care was provided to 809 non-Jordanian children with cancer, with Syrians, Palestinians, Iraqis, and Yemenis being the most common nationalities. The most common cancers were solid tumors, leukemia, brain tumors, and lymphoma.

The ongoing war in Syria and the subsequent influx of refugees have placed significant pressure on the local health system in Jordan. Cancer and cancer therapy are increasingly recognized as significant burdens due to their economic, social, and health implications (15), particularly in low- and middle-income countries (LMICs) like Jordan.

The report also highlights the inadequate funding allocated for cancer treatment for displaced patients in the Middle East, similar to other reports (16). The majority of pediatric applications for cancer treatment made to the UNHCR in Jordan are approved, compared to less than half of adult applications (16). The main justification for application rejection is a poor prognosis.

The WHO estimates that 400,000 new cases of childhood cancer are diagnosed annually, with LMICs accounting for 84% of childhood cancer cases (17). Although childhood cancer is highly curable, the costs of care exceed what displaced families can afford (15). The KHCC currently treats the majority (85%) of new childhood cancer cases in Jordan. The report suggests that the number of displaced pediatric cancer patients in Jordan exceeds that highlighted in the report as a significant number remain underdiagnosed or do not seek medical attention due to various reasons.

The report also highlights the success of the collaborative efforts and robust partnership between the KHCC/KHCF and the SJCRH/ALSAC in optimizing the care and providing financial coverage for cancer treatment through the establishment of the HFs (HF1 and HF2) for displaced children. The results show that the updated patient outcomes treated through these funds are favorable and comparable to Jordanian patients. The collaboration between the KHCC/KHCF and the SJCRH/ALSAC was critical for the success of these efforts in addressing the humanitarian crisis in Jordan. Similar results from the region address displaced pediatric cancer patients treated in Lebanon (18) and Turkey (15). Approximately 100 displaced Syrian children are diagnosed with cancer in Turkey annually (15) (the third-highest refugee burden after Lebanon and Jordan, 3.6% of the Turkey population). The government covered 212 displaced children with cancer for full treatment (18) with outcomes similar to Turkish children. Likewise, Saab et al. (18) described the collaboration for displaced children with cancer in Lebanon established through SJCRH/ALSAC support *via* HFs to deliver quality cancer care (full and partial treatment) to 311 non-Lebanese children using prioritization strategy with encouraging outcomes (78%).

Our report has several limitations including brief follow-up time (35% of patients are on active therapy), the fact that the lack of follow-up data with a significant number of displaced patients could not be traced, and the limited number of patients in each diagnosis subsets, which limits comparisons with expected outcomes. Additionally, precise long-term outcome data on displaced children with cancer treated by KHCC/KHCF funds could not be accurately established since a significant number of patients could not be traced after completing therapy. Additionally, our study may not accurately reflect the entire number of displaced children in Jordan as it includes only refugees registered with the UNHCR. Moreover, survival among displaced children with cancer is high; this should be interpreted with caution. Likely explanations include referral bias (accepting a younger age group, less metastatic, a better prognosis, and less sick patients), and the possibility that mortality registration is missed for some patients as they do not have a national identification. Moreover, it was shown that Jordanian patients have more cases diagnosed in the late stage; this is mostly explained by selection bias.

Despite these limitations, this report offers the only data addressing the burden and characteristics of displaced children with cancer in Jordan, a population that is challenging to enumerate, evaluate, and follow-up over time. The underlying cancer diagnosis for which displaced children are treated in Jordan is also described with cost data given, which can provide the baseline information for the international community to assess and prioritize funding and respond with well-informed and structured programs and interventions. Moreover, these initiatives are well aligned with the Global Initiative for Childhood Cancer led by the WHO and SJCRH aiming to integrate childhood cancer into national cancer control planning (19).

In conclusion, this report highlights the significant burden of childhood cancer among displaced children in Jordan and the inadequate funding allocated for cancer treatment for displaced patients in the Middle East. It also highlights the success of collaborative efforts, partnerships, and financial coverage provided for the treatment of displaced children with cancer in Jordan through the establishment of the HFs. These insights can inform further multistakeholder-coordinated action plans for addressing the humanitarian crisis in Jordan and other similar contexts.

## Data availability statement

The raw data supporting the conclusions of this article will be made available by the authors, without undue reservation.

## Author contributions

RR: patient enrollment, budgeting, data collection and analysis, and the drafting of the manuscript. IS: patient enrollment, program budgeting, data collection and analysis, and the drafting of the manuscript. SJ: programs, budgeting, the review of the manuscript. MN: patient enrollment, budgeting, and the editing of the manuscript. CR-G: programs, budgeting, and the review of the manuscript. AM: programs, budgeting, and the review of the manuscript. All authors contributed to the article and approved the submitted version.

## Supplementary material

Supplementary Figure 1 Flow chart of children with cancer treated at KHCC and fully registered on the KHCC- Cancer Registry between January 2011- June 2022; these include Jordanian and displaced children with cancer. The chart illustrates the different funds covering the cost of treatment of displaced children with cancer.
