# Supplementary figures and images for: The burden and scope of childhood cancer in displaced patients in Jordan: The King Hussein Cancer Center and Foundation Experience
Source: Front Oncol. 2023 Mar 24;13:1112788. doi: 10.3389/fonc.2023.1112788 (PMC10080160; doi:10.3389/fonc.2023.1112788)

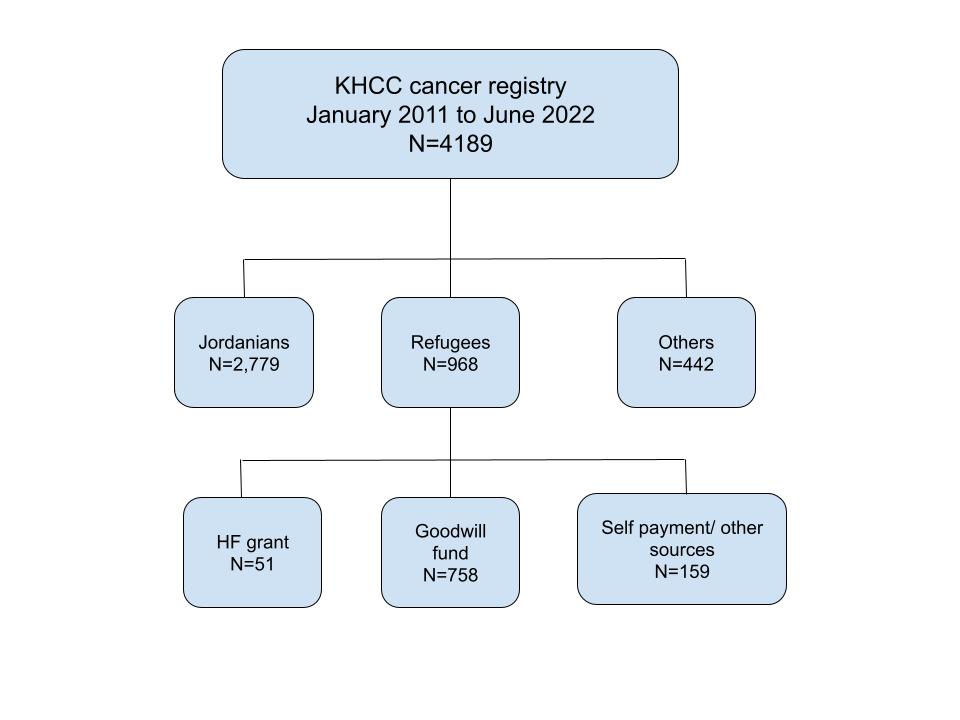

Supplement: Supplementary Figure 1 — Flow chart of children with cancer treated at KHCC and fully registered on the KHCC- Cancer Registry between January 2011- June 2022; these include Jordanian and displaced children with cancer. The chart illustrates the different funds covering the cost of treatment of displaced children with cancer. [file Image_1.jpeg]
